# Application of Hybrid Absorptive–Diffusive Panels with Variable Acoustic Characteristics Based on Wooden Overlays Designed Using Third-Degree-of-Freedom Bezier Curves

**DOI:** 10.3390/ma17225421

**Published:** 2024-11-06

**Authors:** Bartlomiej Chojnacki, Kamil Schynol, Klara Chojnacka

**Affiliations:** 1 Department of Mechanics and Vibroacoustics, AGH University of Krakow, Mickiewicza Av. 30, 30-059 Cracow, Poland; 2 Form At Wood sp. z o.o., Technologiczna 2, 45-839 Opole, Poland

**Keywords:** sound absorbers, diffusion, reverberation chamber, perforated panels, variable acoustic, ISO 354, ISO 11654, small-room acoustics

## Abstract

This manuscript describes the application of novel hybrid acoustic panels with variable acoustic properties that could be used in the design process. Despite the significant growth in the modern acoustic absorbing and diffusing panel sector in recent years, there is still a need for sustainable and original designs that will fit standard interior design trends. The most significant requirement is satisfying the design needs of variable acoustic venues. The availability of acoustic panels with variable properties is minimal, as most designs are based on textiles in the form of rolling banners; therefore, there is no market diversity. The current paper presents an original solution for a novel perforated wooden panel based on third-degree-of-freedom curves. Due to the possibility of exchanging the front panel, the acoustic surface can be varied and adjusted to the room considering different requirements for the acoustic climate, for example, by modifying the attenuation range from low to mid–high frequencies. The novel panels have unique esthetic properties with functional acoustic features regarding sound diffusion and absorption. In this paper, sound absorption and diffusion measurements will be presented for the different variants of the panels, presenting the option to modify the parameters to adjust the panel’s features to the room’s needs. In situ acoustic measurements in a laboratory were conducted to test the variable acoustic panels’ influence on the room’s acoustic parameters, such as T30 and C80. In summary, the advantages of this kind of design will be discussed, alongside the possible impact on modern construction materials’ utilization in architecture.

## 1. Introduction

The acoustic panel market is constantly changing to adjust to the requirements of modern interior design and architecture. For years, it has been evident that standard panels offer limited room for improvement in acoustic quality and economic efficiency, as they rely on basic porous materials [1]. The common challenges are the sustainability and esthetics of acoustic materials. Most of the recent research on sound-absorbing or sound-diffusing structures has been in the field of artificial metamaterials [2,3,4], which has led to significant reductions in the depth of used materials. However, their complexity and the lack of efficient production methods make them challenging to apply on more extensive surfaces, as required for specific room acoustics [5]. Within the state of the art and market review, it should be stated that two essential problems are still present:

- Esthetics side—as most acoustic absorbers are plain, uniformly formed surfaces, such as textile-covered fiberglass or acoustic perforated panels made of MDF or gypsum board, including sound absorption in the modern design process is complicated and requires considerable flexibility from architects [6,7].
- Material sustainability—although there have been some advancements in this field, especially in novel porous materials [8,9,10], the most common method for achieving partially sustainable properties is mixing raw materials, which can be achieved by recycling and developing well-known materials such as polyester fibers or fiberglass. Using materials such as sustainable wood or pure natural fibers is uncommon and expensive. Some attempts to use natural-origin materials have been made, such as the use of sheep wool [11,12]; however, it has been proven that pure, natural materials do not fit architectural requirements as they do not provide sufficient flame retardancy and hygiene properties.

Considering the above arguments, it is clear that there is still a need for the development of traditional acoustic materials to support market diversification. Regardless of the standard materials, the market size is even smaller when considering materials with variable acoustic properties. Applying changes quickly to multipurpose venues such as theaters, conference rooms, or concert halls due to the need to meet multiple functions is a common problem in architecture and acoustic design and is a significant need in the current market [13,14]. So-called multipurpose rooms are commonly built, especially in smaller cities where there is no need for separate rooms for all of the required performance functions [15,16]. Typically, they utilize textile-based materials, such as roller banners, as they are efficient and cheap, but they do not offer any improvements in terms of their visual appeal, and there is no space to change this feature. Therefore, architects are demanding new materials and options for use in original and modern interior design.

This research explores the potential applications of modified acoustic panels featuring third-degree-of-freedom wooden overlays, providing flexible acoustic properties. The rest of the paper is formatted as follows: In Section 2, we present the research motivation in detail and the current state of the art in the field of novel acoustic panels. Section 3 presents measurements of the laboratory space for several configurations of novel panels. In Section 4, in situ example room acoustic measurements are conducted, utilizing the possible changes applied in the panels. Section 5 covers the summary and defines the outcome of the research.

## 2. Research Motivation and State-of-the-Art

The fundamental motivation for the current research was adapting novel acoustic solutions—perforated wooden panels—to variable acoustic needs for modern interior design requirements. As has been proven, there is still a great need to develop new solutions to meet modern interior design requirements, especially if adjustable acoustics are a required feature.

### 2.1. Overview of Available Solutions for Variable and Hybrid Acoustic Properties

**Figure 1:**
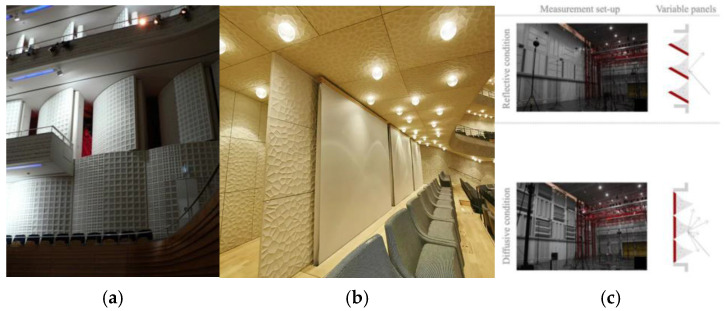
Examples of the variable acoustic in the concert halls: (**a**) reverberation chambers and steerable reflection surfaces used for the reflection redirection and reverberation increment [25], (**b**) acoustic roller banners by Gerriets used as sound-absorbing textiles placed 20 cm from the wall [26], (**c**) rotating diffusive–absorptive–reflecting surfaces in the IRCAM hall used in the experiments of diffusing surface perception [27].

The most typical representations of variable acoustics are shown in Figure 1. Most previous designs were focused on rolling textiles and so-called acoustic banners. Their primary function is to reduce the reverberation time by providing additional absorption, with the textiles mounted by the air gap next to the wall [17,18], but typically, they do not offer modifications of other parameters. They are cheap and efficient and are adopted primarily in concert halls where electroacoustic equipment support and a shorter reverberation time are required. However, the banners do not offer the option for reflection redirection or any diffusive features. The generation of additional reflections to increase the reverberation time is possible through so-called coupled reverberation chambers, which can be opened or closed according to need. They allow for a significant increase in the reverberation time, which is positive if the venue is supposed to serve as a concert hall [19,20]. They are efficient solutions with numerous applications. Unfortunately, they are costly to build and difficult to include in the concert hall space as they take up significant space in the interior [21]. There is also a significant need for hybrid panels that combine diffusion and absorption properties. So far, this kind of panel is unavailable on the market, and most similar solutions are based on the installation of complicated mechanisms [22,23,24].

### 2.2. Hybrid Panels Based on the Wooden Overlays in Third-Degree Curves

**Figure 2:**
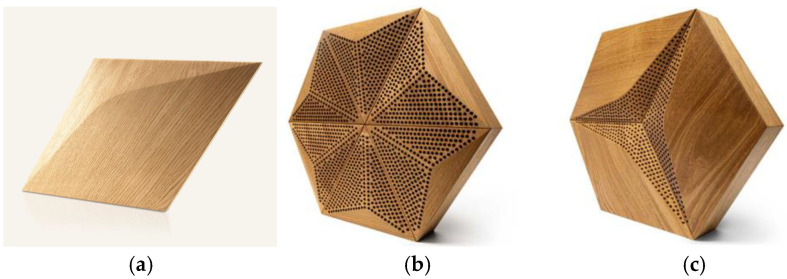

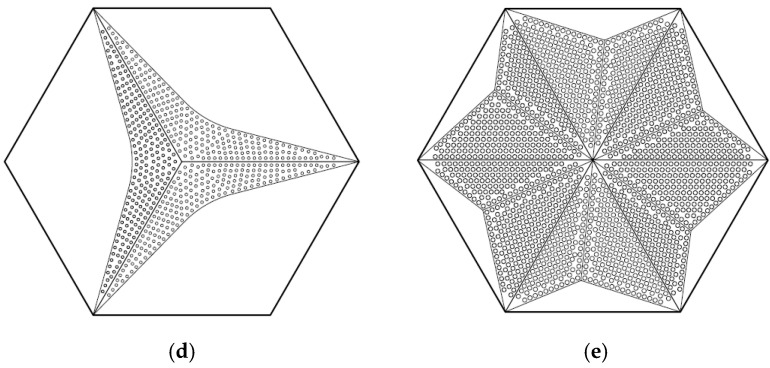
Novel acoustic panels investigated in current research: (**a**) plain overlay CM-D used for replacing the parts of acoustic panels, (**b**) hexagonal-shaped perforated wooden panel in the P-A option, (**c**) hexagonal-shaped perforated wooden panel in the CM-D option, (**d**) the top view of the CM-D overlay with the perforation pattern sketch, and (**e**) the top view of the P-A overlay with the perforation pattern sketch.

The current study utilizes the novel design of wooden perforated panels [28]. The basis of the panel is a wooden overlay designed in a 3D shape with third-degree curves and a depth change between 0 and 30 mm. The perforation is provided with drills of 6 mm diameter in a unique pattern for each type of overlay. To achieve sustainability, the FSC (Forest Stewardship Council)-certified type of wood is used based on recycled materials and can be reused. The innovation included in a new version of the panels is the possibility of fast overlay exchange, which allows the modification of the front panel shape to change the perforation ratio and balance of the absorptive–diffusive properties of the panels. The product’s exterior design is maintained thanks to replacement with the same shape overlays without the perforation patterns. The examples of the panels used in current research are shown in Figure 2.

The proposed solution incorporates two novel capabilities into the traditional absorptive panels: diffusion properties and the ability for panel modification by exchanging the top overlays for non-perforated overlays and modifying the absorption curve. The novel shape of the proposed panels complies with modern interior design requirements but also leads to difficulties in introducing acoustic properties into this kind of structure and, thus, in maintaining both the visual and acoustic features of the product.

## 3. Acoustic Property Measurements

The laboratory measurements of different panel variations will be presented in the current section to demonstrate their universal and flexible characteristics that can be adjusted to the desired function and the designed venue. As calculation techniques typically cannot accurately determine the final acoustic material properties [29,30,31], all desired parameters were measured in dedicated laboratory spaces at the AGH University of Krakow.

### 3.1. Sound Absorption in a Reverberation Chamber

**Figure 3:**
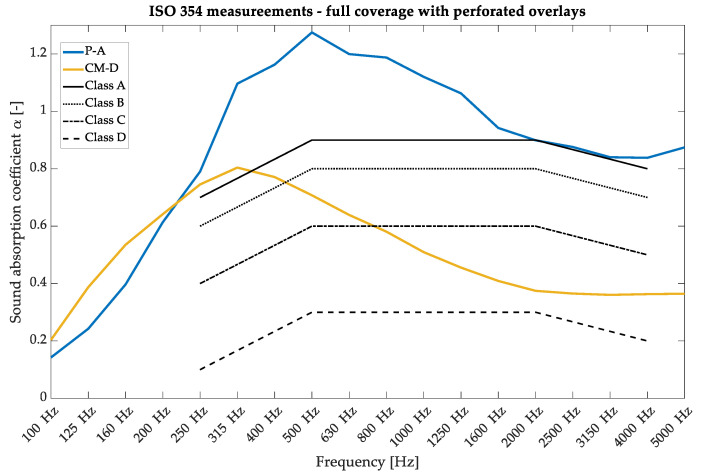
Sound absorption coefficient measurements in a reverberation chamber under ISO 354 requirements for the wooden perforated overlays—variants of 100% P-A and 100% CM-D with 5 cm of fiberglass were used.

The first parameter investigated was the random incidence sound absorption coefficient, typically written as α in scientific papers. The measurements were performed in a reverberation chamber at AGH University following the ISO 354 [32] requirements for plain materials [32]. The measurements were performed using the sine sweep method, registered using six GRAS 46AQ microphones, and two omnidirectional sound sources emitted the signal. The sample surfaces were fixed at 10.4 m^2,^ and additional frames were used to limit the edge effects [33,34]. Figure 3 shows the sound absorption coefficient for the P-A and CM-D types with a composition of 100% overlay and 5 cm of fiberglass used as an absorptive core. Panel development processes have been comprehensively described in previous research [28] and were used as a reference for variable acoustic properties in our research. The sound absorption results in Figure 3 exceed the value of 1.0, a common problem in measurements inside a reverberation chamber [35,36]. This phenomenon is caused by several reasons, such as the finite size of the sample, the inaccurate diffuse field inside the reverberation chamber, and the so-called edge effect. However, in most cases, values that exceed 1.0 are treated at the maximum level of 1.0 and then applied to room acoustic models [37,38].

**Figure 4:**
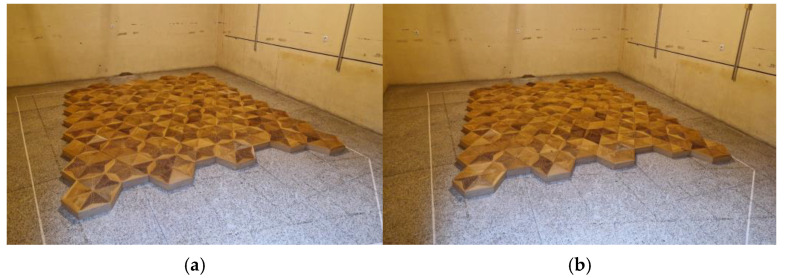
Samples set up in a reverberation chamber in perforated and solid panels merging experiments: (**a**) sample with 66% of perforated overlays and 33% of solid ones; (**b**) sample with 33% of perforated overlays and 66% of solid ones.

Standard perforated overlays could be replaced with solid ones with a reduced perforation rate and different absorption properties to vary the acoustic parameters. The panel configuration used in this research was selected considering the physical possibility of adjusting the overlays by replacing the complete overlay with another one in the same shape but made of PET boards, for example. The other consideration when selecting the configurations was that the visual aspects should be kept and irregular panel division should be avoided. The selected configurations are examples of the possible uses and allow for the comprehensive assessment of the possible changes in acoustic properties. They could then be used to determine other similar configurations. In the next step, P-A-type panels were evaluated to assess the changes in absorption after replacing some overlays with solid ones. The samples used for this configuration setup are shown in the reverberation chamber in Figure 4.

**Figure 5:**
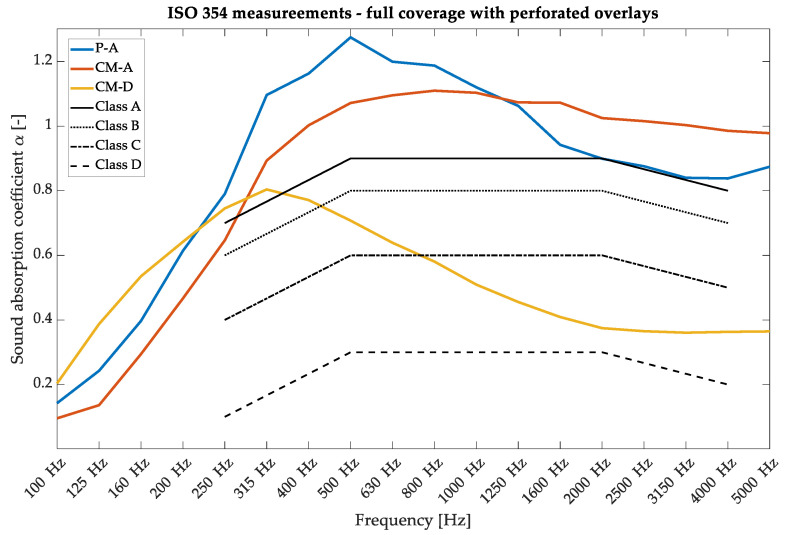
Sound absorption coefficient measurements in a reverberation chamber for the wooden perforated overlays—variants of 100%/66%/33% of P-A overlays with 5 cm of fiberglass were used.

The results of sound absorption measurements shown in Figure 5 display the achieved variability in the results. A significant reduction in high-frequency absorption can be achieved by replacing some of the overlays with solid ones. However, low-frequency absorption is still high. This could be a valuable feature to compensate for features such as additional furniture, which is helpful in theaters when additional scenography is introduced [18]. The change in the balance between high and low frequencies is also helpful regarding human absorption in small-room or concert hall acoustics [39].

**Figure 6:**
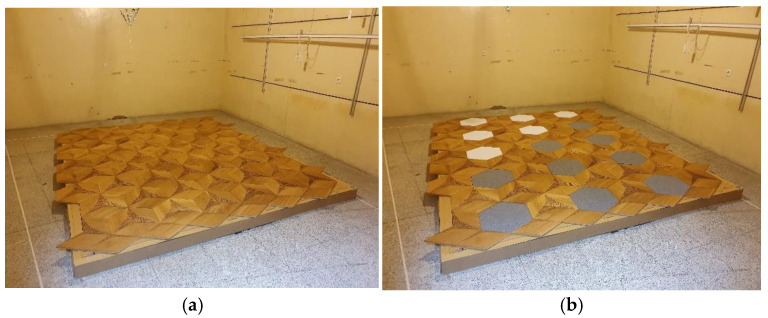

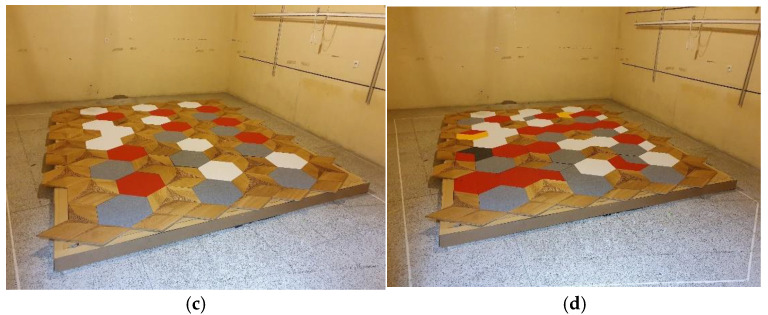
Samples set in the reverberation chamber in perforated and PET-based panels merging experiment conducted under ISO 354 standards requirements: (**a**) sample with 100% perforated overlays and 0% PET; (**b**) sample with 80% of perforated overlays and 20% PET; (**c**) sample with 60% perforated overlays and 40% PET; and (**d**) sample with 20% perforated overlays and 80% PET.

The exchange of perforated overlays with solid ones is a valuable and easy method that could be applied to individual panels, which allows for a fast change in the surface’s acoustic properties without affecting the panel’s esthetics. Another proposed solution is mixing standard overlays with porous materials, such as PET fiberboards. These boards have a typical surface density of 2 kg/m^2^, a 9 mm depth, and a flow resistivity of around 30,000 Pa·s/m^2^. Their significant advantage is their sustainability, as they are based on recycled PET bottles. They offer significant advantages as a material with a sustainable surface composition and wooden overlays made from FSC wood. We designed other configurations using CM-D panels and 10 cm of fiberglass as an absorbent core to investigate the possibilities of merging PET fiberboards with wooden perforated overlays. Examples of these configurations are shown in Figure 6.

**Figure 7:**
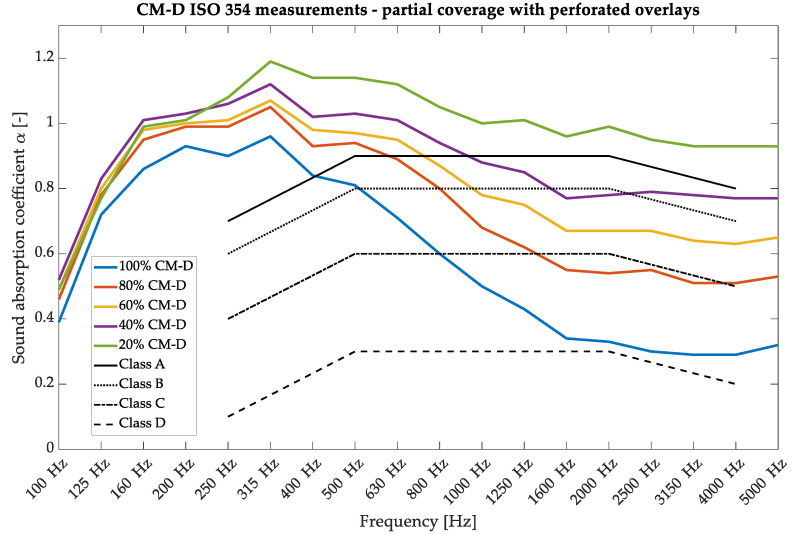
Sound absorption coefficient measurements in a reverberation chamber for the wooden perforated overlays—variants of 100%/80%/40%/20% of CM-D overlays with 10 cm of fiberglass were used.

The results of absorption measurements for the above configurations are shown in Figure 7. Replacing part of the wooden perforated overlays with porous PET panels significantly improves absorption, especially in the high-frequency range; therefore, these panels exhibit a contrasting behavior when used as overlays compared to the P-A panels. It was proven that even adding a small amount of PET fiberboard (20%) increases the sound absorption by 0.25 in the high-frequency range and also maintains the low-frequency priority in the absorption curve. With the lower percentage of PET panels (ample coverage with perforations), a resonant-type character of the curve is observed, with the maximum value at 315 Hz, which is determined by the configuration of the holes used during perforation manufacturing. With an increasing percentage of PET panels used, the absorption curve shape resembles that of typical broadband-absorbing panels.

To summarize the possible changes in the novel panels’ absorption properties, Table 1 shows the used variant names and compares their weighted sound absorption coefficients and absorption classes, which were calculated using the requirements of ISO 11654 [40]. The α_w_ values were calculated according to the standard requirements by using the reference curve shifting and the maximum deviation from the reference curve calculation. The absorption class is a critical parameter for architects as it allows for the fast assessment of product features and easy inclusion into the building’s technical datasheet. However, in most specialized acoustic venues, different absorption classes are required to adjust the final absorption type to the given venue’s needs. The current research proved that exchanging some of the perforated overlays and the base versions of the considered panels may allow for a significant change in the sound absorption class between classes D and A while using the same wooden overlay type. This offers unique possibilities for acoustic designers and architects when including those panels in the design process.

### 3.2. Sound Diffusion in an Anechoic Chamber

**Figure 8:**
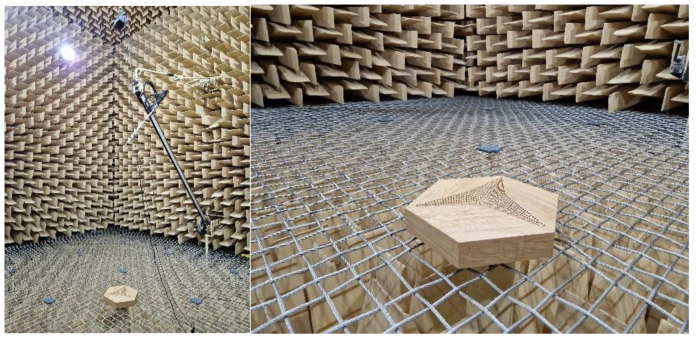
Experimental measurements of the diffusion in an anechoic chamber for hybrid panels with CM-D-type wooden overlay.

The hybrid panels were designed to utilize both the absorption and diffusion properties of their surface. Thanks to the 3D shape of the wooden overlays and the height change across the panel (between 0 and 30 mm), diffusion is achieved. This is because the arc-shaped surfaces could be treated as diffusive panels [41]. Measurements were performed in an anechoic chamber with the ISO 17497-1 [42] standard requirements to assess the real-life diffusion of the proposed novel solution. A photograph of the experimental measurements for the measured panels is shown in Figure 8.

**Figure 9:**
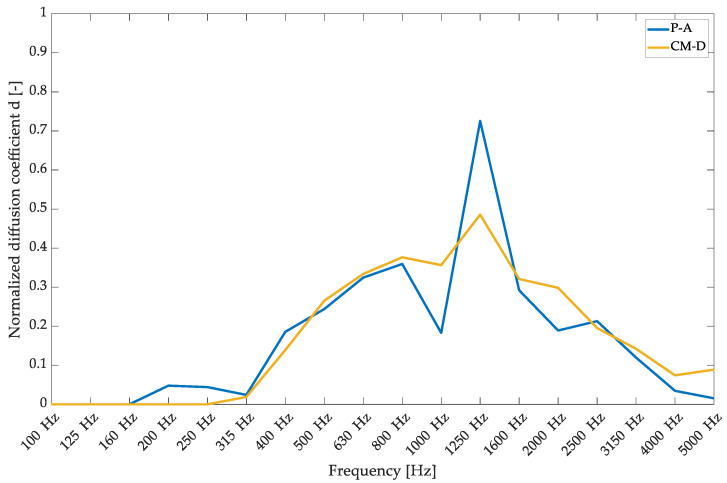
Diffusion coefficient measurements in an anechoic chamber for the proposed hybrid panels with CM-D and P-A wooden overlays.

The results of the normalized diffusion coefficient measurements are shown in Figure 9. Both types of overlays achieved a peak normalized diffusion coefficient of 0.3–0.4 in the frequency range of 630–2000 Hz. However, the diffusion values were not very high. It is important to note that typical diffusion-dedicated products achieve a normalized coefficient of 0.7–0.75. However, sometimes, to improve the results, a non-normalized curve is presented, which could be viewed as improper data presentation [29,43]. The results achieved for the presented hybrid solution should be rated as average.

## 4. In Situ Acoustic Measurements

**Figure 10:**
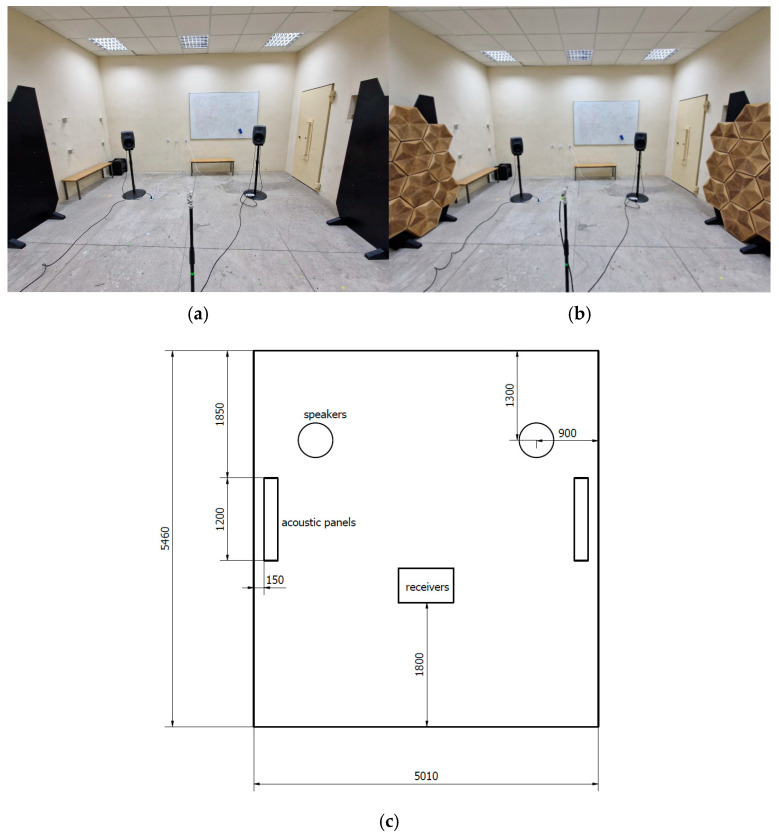
Photographs of the room used for in situ verification of hybrid panel application: (**a**) without the hybrid panels on the side walls; (**b**) with the panels installed. (**c**) A sketch of the measurement situation with the location of the source and receiver (Unit: mm).

The final part of the proposed research was the in situ verification of the hybrid panels in a typical small-room acoustic environment. The measurements were performed in a 26 m^2^ small-room acoustic laboratory at the Department of Mechanics and Vibroacoustics at AGH University of Krakow. A photograph of the measurement room is shown in Figure 10. The chosen measurement setup and facility provided a controlled environment for accurate and reliable data collection. Two stands with hybrid panels were placed at the points of first-order reflections to address the most important acoustic faults in the acoustics of this small room. This allowed for the assessment of the acoustic performance of the wooden overlay panels, considering their real-world application and potential ability to improve room acoustics.

This research was conducted following the guidelines specified in ISO 3382 [44]. The abbreviation from the standard requirements was included as a replacement for omnidirectional sound sources with a directional studio monitor, which better reflects the actual acoustics in the small room and is more suitable for the considered situation. The results were analyzed using B&K 7841 Dirac 5.0 software, which enabled the calculation of fundamental acoustic parameters, such as reverberation time T30 and sound clarity C80. The following equipment was used:

⯀ GRAS 12AL microphone power supplies (Holte, Denmark);
⯀ An RME Fireface UFX measurement card (Haimhausen, Germany);
⯀ A Lenovo ThinkPad portable computer (Morrisville, NC, USA) with B&K 7841 Dirac 5.0 software;
⯀ A GRAS 46 AE ½″ microphone (Holte, Denmark);
⯀ A Genelec 8030 sound source (Iisalmi, Finland).

**Figure 11:**
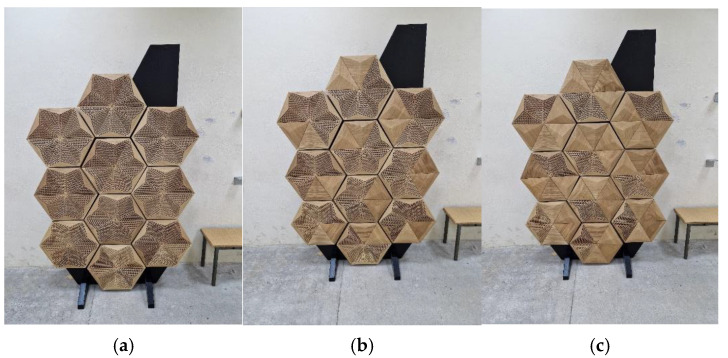
Variants of the variable acoustic hybrid panels used in the in situ research: (**a**) 100% perforated overlays, (**b**) 66% perforated overlays, and (**c**) 33% perforated overlays.

The measurement data were processed and analyzed using established methodologies and algorithms incorporated in B&K Dirac software. To validate the influence of hybrid panels and their variable acoustic properties, two dedicated stands were used with 10 panels on each of them. The overall surface of the acoustic panels was 2.84 m^2^. The panels were used in three previously measured configurations—perforated P-A wooden overlays were partially replaced with solid ones, as shown in Figure 11.

**Figure 12:**
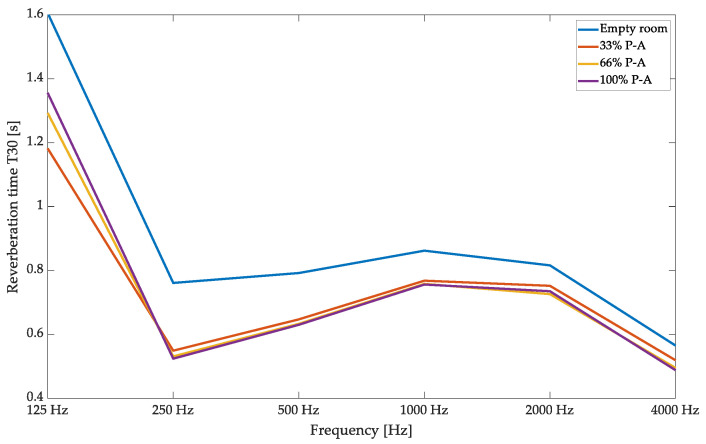
Validation of hybrid variable acoustic panels in a small room—reverberation time analysis.

The first parameter considered in the current research was the reverberation time; measurement results are presented in Figure 12. Including the panels (2.84 m^2^) in the room significantly reduced the reverberation time, and placing them at the first reflection points allowed for the best possible performance. The variable acoustic mechanism allowed for slight tuning of the affected frequency range; low-frequency-range modifications were the most effective. Despite the slight changes and relatively small surface of the panels relative to the overall room surface, the panels in the 33% case achieved a T20 of 1.15 s, which translates to a 0.45 s reduction, which is a significant reduction considering the surface of panels used. The high- and mid-frequency range attenuation remains unaffected, with the highest change in T30 of 0.05 s found at this parameter’s border with the JND value. Therefore, the proposed solution’s variable acoustic properties allow for fine-tuning in the room, especially in the low-frequency range. The findings are important in comparison with ISO 354 measurements presented in Section 3, as we can expect the most significant changes to occur in the high- and mid-frequency ranges, as the absorption coefficient changes are the highest in this range. However, due to specific characteristics of small-room acoustics, the in situ measurements displayed the opposite trend.

**Figure 13:**
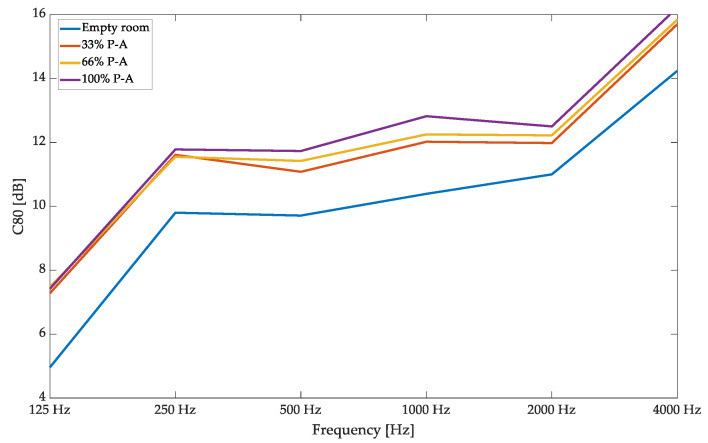
Hybrid variable acoustic panel validation in small-room acoustic measurements—sound clarity analysis.

Despite the reverberation time, the C80 parameter was used in the analysis to describe the perception of music clarity and the balance between the early and late parts of the decay curve registered in the room. The results are presented in Figure 13. In this case, the changes in the variable acoustic properties of the panels, achieved by exchanging the overlay, are correlated with the received absorption curve changes noted in the sound absorption measurements in the reverberation chamber. Overlays with a smaller percentage of perforation (33% or 66%) had more negligible absorption. Therefore, their reflection coefficient was higher, which resulted in the redirection of the acoustic energy into the later part of the decay curve, resulting in a smaller C80 value. However, the effect of the change in the percentage of perforation was negligible in the low-frequency range, which is in agreement with the changes in the previously measured absorption curve.

In the current study, only side first-order reflections were studied, with a total of 2.84 m^2^ of panels. It is important to note that if more panels were included, the manipulation of the room’s acoustic properties could potentially be significantly improved, and the panels could induce broader changes in the T30 and C80 values. On the other hand, in bigger venues, the panels’ surfaces should be adjusted to achieve a variable acoustic effect. It is essential to verify the variable acoustic panel surface that is required for the given performance function of the venue each time. Simulations should be conducted using room acoustic simulation methods such as raytracing.

## 5. Summary and Conclusions

This article presented the concept of using hybrid absorption–diffusive panels in small rooms to modify the properties of the acoustic field considering the room’s desired function. A novel type of wooden 3D panel was used using the top panel’s overlay-exchange function, which allowed for significant changes in their absorption curves. Exchanging the overlay allowed for a modification of the sound absorption class between the D and A values depending on the overlay configuration used.

The properties of the panels used in the research were confirmed in the ISO 354 and ISO 17497-1 standard measurements in the laboratory and then tested in situ in a model small-room acoustic venue. The results proved that the changes to the front panels (changing the perforated panels to solid panels) could significantly impact the sound field in the room and allow for fine-tuning the room acoustics for the desired function. Fewer perforated overlays significantly reduce the reverberation time, while panels with higher percentages of perforated surfaces had increased C80 values, resulting in improved musical clarity. Despite the small surface area of the panels used in the measurements, the changes measured were significant and above the just-noticeable difference defined for these parameters.

The development of these novel variable acoustic panels allows for the further development of variable acoustic spaces that comply with modern acoustic requirements. Nowadays, options other than acoustic banners and reverberation chambers have been developed, and wooden acoustic wall panels are easier to apply to large surfaces than complicated construction solutions.

## Figures and Tables

**Table 1:** Single-value parameters for the measured hybrid panels’ configurations.

Variant Name and % of Perforation	α_w_ ISO 11654	Absorption Class ISO 11654
33% P-A	0.45 (LM)	D
66% P-A	0.75 (M)	C
100% P-A	0.95	A
20% CM-D 80% PET	1.00	A
40% CM-D 60% PET	0.80	B
60% CM-D 40% PET	0.70	C
80% CM-D 20% PET	0.60 (LM)	C
100% CM-D	0.40 (LM)	D

## Data Availability

The raw data supporting the conclusions of this article will be made available by the authors on request.
